# Hornerin promotes tumor progression and is associated with poor prognosis in hepatocellular carcinoma

**DOI:** 10.1186/s12885-018-4719-5

**Published:** 2018-08-13

**Authors:** Shun-Jun Fu, Shun-Li Shen, Shao-Qiang Li, Yun-Peng Hua, Wen-Jie Hu, BeiChu Guo, Bao-Gang Peng

**Affiliations:** 1 Department of Liver Surgery, First Affiliated Hospital, Sun Yat-sen University, Guangzhou, 510080 China; 2 Department of Hepatobiliary Surgery, Zhujiang Hospital, Southern Medical University, Guangzhou, 510280 China; 3 Department of Microbiology and Immunology, Hollings Cancer Center, Medical University of South Carolina, Charleston, 29425 USA

**Keywords:** Hornerin, Hepatocellular carcinoma, Tumor progression, Prognosis, AKT

## Abstract

**Background:**

The function of hornerin (HRNR), a member of the S100 protein family, is poorly clarified in the development of human tumors. The role of HRNR in hepatocellular carcinoma (HCC) progression is investigated in the study.

**Methods:**

The expression levels of HRNR were assessed in tumor samples from a cohort of 271 HCC patients. The effect of HRNR on proliferation, colony formation and invasion of tumor cells was examined. We further determined the role of HRNR in tumor growth in vivo by using xenograft HCC tumor models. The possible mechanism of the HRNR promotion of HCC progression was explored.

**Results:**

We found that HRNR was overexpressed in HCC tissues. The high expression of HRNR in HCCs was significantly associated with vascular invasion, poor tumor differentiation, and advanced TNM stage. The disease-free survival (DFS) and overall survival (OS) of HCC patients with high HRNR expression were poorer than those in the low HRNR expression group. HRNR expression was an independent risk factor linked to both poor DFS (HR = 2.209, 95% CI = 1.627–2.998,*P* <  0.001) and OS (HR = 2.459,95% CI = 1.736–3.484, *P* <  0.001). In addition, the knockdown of HRNR by shRNAs significantly inhibited the proliferation, colony formation, migration and invasion of HCC tumor cells. HRNR silencing led to the decreased phosphorylation of AKT signaling. Notably, tumor growth was markedly inhibited by HRNR silencing in a xenograft model of HCC.

**Conclusions:**

HRNR promotes tumor progression and is correlated with a poor HCC prognosis. HRNR may contribute to HCC progression via the regulation of the AKT pathway.

## Background

Hepatocellular carcinoma (HCC) is one of the common malignant diseases and the second most common cause of cancer-related death worldwide [1]. Furthermore, the incidence of HCC has also been on the rise. Liver resection or transplantation is considered effective treatments for HCC. Despite improvements in diagnosis and therapeutic methods for HCC, the prognosis remains poor. Therefore, the identification of novel targets to improve the clinical management of HCC is essential.

The gene of hornerin gene (HRNR) is clustered on the chromosome region 1q21 [2]. This gene was first discovered in the mouse embryo epidermis, and was detected in the skin, tongue, oesophagus and proximal stomach of adult mice. HRNR is the member of S-100 fused protein family, which has a Ca^2+^ binding EF-hand domain at the N-terminus followed by a spacer sequence and an extensive repetitive domain rich in glycine and serine [3]. S100 proteins are reportedly involved in the physiological and pathological processes such as the regulation of protein phosphorylation, inflammatory and immune reactions, calcium homeostasis, transcription factors, cytoskeleton components, cell proliferation, differentiation and death [4]. Differential expression of the S100 family proteins has been found in many tumors [5–7]. HRNR was reported to be involved in breast cancer development and malignant transformation [8]. Previously, we found that the expression of HRNR in HCC tissues was elevated via proteomic analysis [9]. However, the roles of HRNR in the development of HCC have not been characterized. The purpose of the study was to define the expression levels of HRNR in HCC patients and its involvement in HCC progression.

## Methods

### Patients and tissue sample specimens

Total of 271 HCC patients was involved in the study. The snap-frozen tumors and corresponding peri-cancerous tissues were collected during liver resection at the Department of Hepatobiliary Surgery, the First Affiliated Hospital of Sun Yat-sen University from January 2006 to December 2008. There was no gender discrimination in the treatment offered (surgery) to patients referred for HCC to our institution. The study was approved by the Ethics Committee of the First Affiliated Hospital of Sun Yat-sen University. All patients signed informed consent. The tumor stages were assessed according to the tumor-node-metastasis (TNM) system of the 2010 International Union Against Cancer by the American Joint Committee. The histological grade of tumors was determined by the Edmondson Steiner grading system [10]. Postoperative patient follow-up was implemented as previously described [11, 12]. The durations of disease-free survival (DFS) and overall survival (OS) were defined as previously described [11, 12]. The last follow-up date was December 31, 2013.

### Cell lines and cell culture

The human HCC cell lines HepG2 (Catalogue Number: HB-8065™), Hep3B (Catalogue Number: HB-8064™) and PLC/PRF/5 (Catalogue Number: CRL-8024™) were purchased from the American Type Culture Collection (ATCC; Rockville, MD, USA). The human HCC cell line Huh7 (Catalogue Number: JCRB0403) was purchased from the Japanese Cancer Research Bank. The human HCC cell lines SMMC-7721 (Catalogue Number: TCHu 52), BEL-7402 (Catalogue Number: TCHu 10), QGY-7703 (Catalogue Number: TCHu 43) and normal liver cell line LO2 (Catalogue Number: GNHu 6) were obtained from Cell Bank (Shanghai, China).

The cells were cultured in low glucose Dulbecco’s modified Eagle media (DMEM), including 10% fetal bovine serum (FBS) supplemented with 100 U/ml penicillin and 0.1 mg/ml streptomycin, and incubated at 37 °C in a humidified atmosphere at 5% CO_2_.

### Cell transfection and stable cell lines construction

The three lentivirus plasmids containing human HRNR shRNAs, vector plasmid pLKO.1 puro, packaging plasmid pHR’8.2 deltaR dvpr and pCMV-VSV-G were purchased from Sigma (St. Louis, MO, USA). These plasmids were extracted according to the protocol (GeneJET Plasmid Maxiprep Kit, Thermo SCIENTIFIC). The lentiviral packaging cells, 293 T cells (CRL-3216™), were transfected with the three lentivirus plasmids containing human HRNR shRNAs or vector plasmid pLKO.1 puro and packaging plasmid pHR’8.2deltaR dvpr and pCMV-VSV-G at 70% confluence with the use of Lipofectamine 2000 (Invitrogen, Carlsbad, CA) to produce the lentivirus. Media containing the lentivirus were added to the target cells for 24 h. After 24 h, the original medium was replaced with fresh medium. The cells containing the shRNA constructs were selected in the medium containing puromycin and were cultured for approximately 2 weeks [13]. The stable cell lines were validated by western blotting.

### Tissue microarray and immunohistochemistry

Tissue microarray construction was done as described [14]. Two 1 mm diameter core biopsies were removed from the donor blocks; then, the samples were transferred to the recipient paraffin block. The immunohistochemical staining (IHC) was used to the avidin-biotin-peroxidase complex method. In brief, after rehydration and heating antigen retrieval, antibodies against human HRNR (1:200, NBP1–80807; Novus) were then used to the slides and incubated at 4 °C overnight. The secondary antibody incubation (Envision Polymer-HRP,anti-Rabbit/Mouse) was then performed at 37 °C for 30 min. The reaction products were visualized with diaminobenzidine staining and Meyer’s haematoxylin counterstaining. Two investigators who did not have any clinical or pathological information regarding the origin of the samples scored the IHC staining. The scores of IHC staining were determined as previously described [15, 16]. Based on the scoring system, HCC tissues were classified as follows: negative, weak, moderate, and strong. The expression levels of HRNR were divided into a HRNR-low group (negative/weak) and a HRNR-high group (moderate/strong). Each sample was scored in a blinded manner by two investigators who did not have any clinical or pathological information regarding the origin of the samples.

### Western blot analysis

The cells were washed twice with ice-cold phosphatebuffered saline (PBS). Proteins were extracted from the cells using RIPA lysis buffer as previously described [17]. The protein concentration was decided with the Bradford reagent (Bio-Rad Laboratories, Hercules, CA, USA) using a bovine serum albumin standard. Equal amounts of total protein were separated on 10% SDS-PAGE gels and subsequently transferred onto PVDF membranes. The membranes were detected overnight at 4 °C with primary antibodies. Western blot bands were detected by electrochemical luminescence (ECL). Protein expression was confirmed by western blot using the following antibodies: hornerin (NBP1–80807; Novus), AKT and p-AKT (Ser473) (9272 and 9271, respectively, Cell Signaling Technology, Danvers, MA,USA), and GAPDH (sc-47,724, Santa Cruz).

### Cell proliferation assay, clone formation assays, cell migration and invasion assays

The cells were placed into a 96-well plate (5000 cells/ well). At different points in time (1, 2, 3, 4, 5 and 6 days), 10 μl of MTT (5 mg/ml, Sigma, USA) was added to each well, and the plate was hatched for an additional 4 h. Then, the medium was exchanged by 150 μl of DMSO and shaken at room temperature for 10 min. The number of viable cells in each well was calculated by the absorbance value (λ = 490 nm).

For the colony formation assay, the cells were placed into a 6-well culture plate (1000 cells/well) and cultured for 2 weeks. The colonies were stained with 1% crystal violet and counted.

For the cell migration assay, transwells (24-well, 8-μm pore size; Millipore, Billerica, MA, USA) were used. A total of 3 × 10^4^ cells in 300 μl DMEM without FBS were seeded in the upper chamber and 800 μl of DMEM with 10% FBS was added to the lower chamber. The upper chamber cells were removed after 48 h incubation and those on the lower surface of the membrane were fixed with methanol, then, the cells were stained with crystal violet, counted (200× magnification), and photographed. The cell invasion assays were performed the same as the cell migration assays, except the transwells were precoated with Matrigel (BD Biosciences, Franklin Lakes, NJ, USA). All above experiments were done in triplicate.

### Xenograft model with human HCC cells

For the xenograft tumor model, 1 × 10^6^ cells were injected subcutaneously into the right upper flank of 5-week-old male BALB/C nude mice. Each group contained 6 mice. Tumor formation in nude mice was monitored over a 32-day period, and the length and width of the tumors were measured every 4 days and their volumes were calculated by the formula: V = 0.5 × length × width^2^. The animal experiment was approved by and performed in accordance with the Ethic Committee on the Use of Live Animals in Teaching and Research at the First Affiliated Hospital of Sun Yat-sen University. The tumor-bearing mice were sacrificed by cervical dislocation.

### Statistical analysis

Statistical analysis was performed with SPSS software (19.0; SPSS, Inc., Chicago, IL). Categorical data were analyzed by the chi-square or Fisher’s exact tests. Cumulative recurrence and survival rates were analyzed using Kaplan-Meier’s method and the log-rank test. Cox’s proportional hazards regression model was used to analyze independent prognostic factors. Variables analyzed by univariate analysis with *P* <  0.05 were involved in the multivariate Cox proportional hazards model. *P* <  0.05 was considered statistically significant.

## Results

### HRNR expression is related with poor prognosis of HCC

**Fig. 1:**
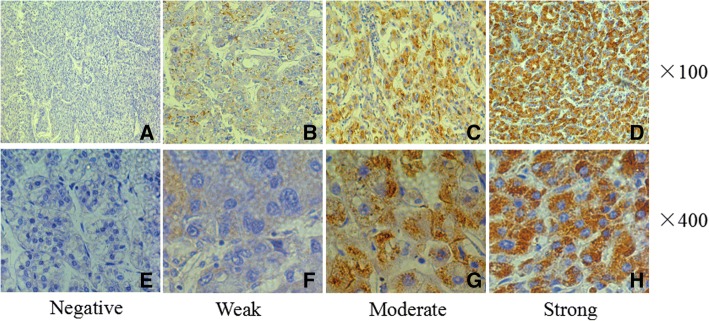
HRNR overpression in human HCC tumor tissues. Immunohistochemistry of HRNR expression in hepatocellular carcinoma (HCC) tissues. HRNR expression in the cytoplasm and membrane is scored as negative (**a**, **e**), weak (**b**, **f**), moderate (**c**, **g**), and strong (**d**, **h**). Original magnification, × 100 (**a**-**d**); × 400 (**e**-**h**)

To explore the role of HRNR in HCC, we analyzed the expression of HRNR in tumor samples from a cohort of 271 HCC patients. Our results showed that HRNR was expressed in 84.5% (229/271) of HCC tissues. High HRNR expression was found in 57.9% (157/271) of patient tissues. HRNR expression was localized mainly in the cytoplasm, with some expression identified on the cell membranes (Fig. 1).

Next, we evaluated whether there was any association of HRNR expression with the clinicopathologic factors of HCC patients. Based on the IHC results, the 271 HCC patients were distributed into two groups: the HRNR-high expression group (*n* = 157) and the HRNR-low expression group (*n* = 114). The results revealed that high HRNR expression in HCC positively correlated with vascular invasion (*P* = 0.002), poor tumor differentiation (*P* = 0.042) and advanced TNM stage (*P* <  0.001); however, the high expression of HRNR in HCCs had no significant correlation with age, gender, HCC family history, hepatitis B, liver function Child-Pugh stage, cirrhosis, tumor size, tumor number, encapsulation and alpha-fetoprotein (AFP) (all *P* > 0.05) (Table 1).

**Table 1:** Relationship between the expression of HRNR and clinicopathological characteristics

Category	Subcategory	Cases	HRNR expression		*P* value
			Low (*n* = 114)	High (*n* = 157)	
Gender	male	240	98	142	
	female	31	16	15	0.826
Age (years)	≤ 50	131	56	75	
	> 50	140	58	82	0.253
HCC family history	Yes	18	8	10	
	No	253	106	147	0.665
HBsAg	negative	32	14	18	
	positive	239	100	139	0.837
Child-pugh stage	A	269	113	156	
	B	2	1	1	0.820
AFP(ng/ml)	< 20	67	22	45	
	≥20	204	92	112	0.078
Edmonson Grading	I-II	212	96	116	
	III-IV	59	18	41	0.042
Tumor Size (cm)	≤ 5	96	47	49	
	> 5	175	67	108	0.089
Liver Cirrhosis	absent	55	27	28	
	present	216	87	129	0.237
Capsulation	capsulated	171	76	95	
	non-caspulated	100	38	62	0.300
Tumor Number	single	185	85	100	
	multiple	86	29	57	0.058
Vascular Invasion	Yes	56	16	40	
	No	215	98	117	0.022
TNM Stage	I-II	152	80	72	
	III-IV	119	34	85	< 0.001

*HRNR* hornerin, *HBsAg* hepatitis B surface antigen, *AFP* alpha fetoprotein

**Fig. 2:**
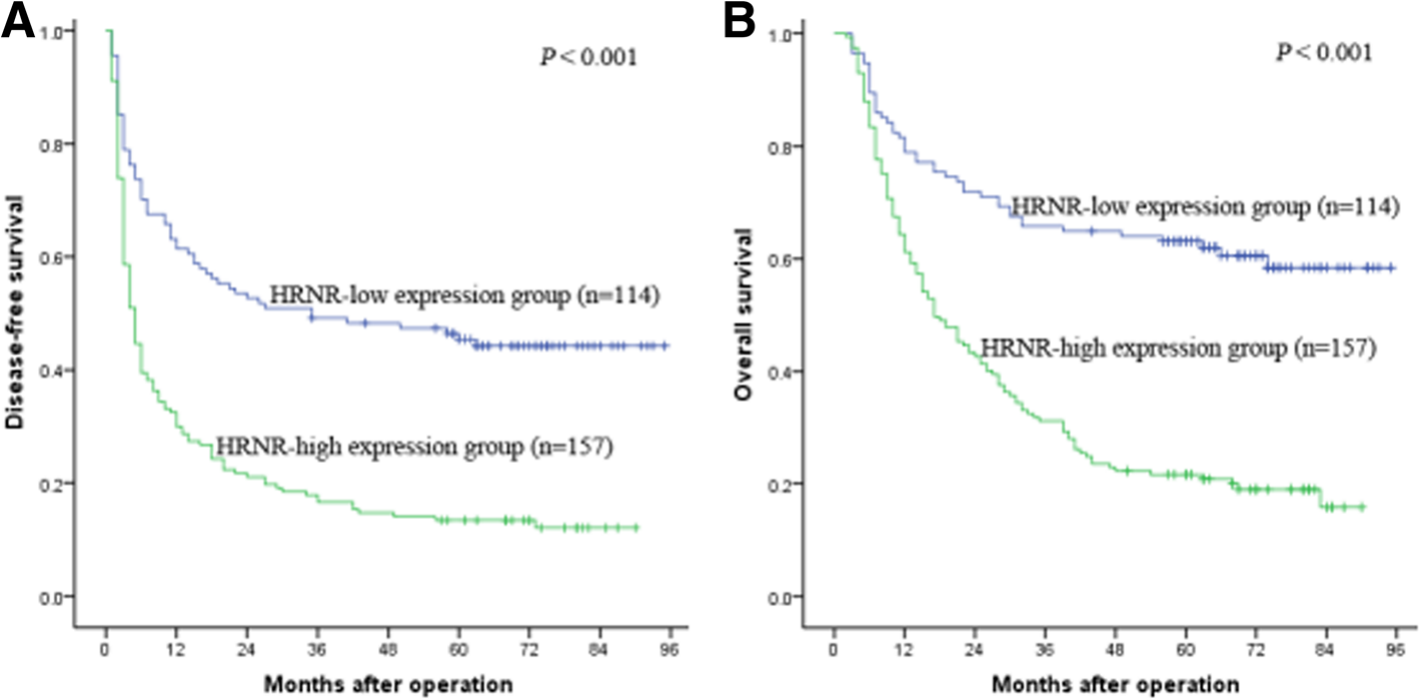
HRNR expression is associated with poor outcome of human HCC patients. Kaplan–Meier survival curves of DFS and OS for the HRNR low expression group (*n* = 114) and the HRNR high expression group (*n* = 157) based on the results of immunohistochemistry. The results show that HCC patients with low HRNR expression have better DFS (**a**) and OS (**b**) than those with high expression of HRNR

We further explored the prognostic value of HRNR expression. We found that the 1-, 3-, and 5-year DFS rates (29.9%, 16.6% and 13.4% VS 61.4%, 49.1% and 45.3%, *P* <  0.001) and OS rates (61.1%, 31.2% and 21.6% VS 78.9%, 65.8% and 63.1%, *P* <  0.001) of HCC patients in the high HRNR expression group were poorer than those in the low HRNR expression group (Fig. 2). Kaplan-Meier analysis indicated that Edmondson grading, tumor size, capsulation, tumor number, vascular invasion, HRNR expression and TNM stage were risk factors for DFS; gender, Edmondson grading, tumor size, capsulation, tumor number, vascular invasion, HRNR expression and TNM stage were risk factors for OS (Table 2). According to the multivariate Cox regression analysis, high HRNR expression was found to be an independent prognostic factor linked to both poor DFS (hazard risk [HR] = 2.209, 95% confidence internal [CI] = 1.627–2.998,*P* <  0.001) and OS (HR = 2.459,95% CI =1 .736–3.484, *P* <  0.001) (Table 3). These findings suggest that high HRNR expression was significantly associated with poor prognosis, indicating a potential role for HRNR in hepatic tumorigenesis.

**Table 2:** Influence of clinicopathological characteristics on patients’ prognosis by Kaplan-Meier analysis

Variables	*n*	DFS				OS			*P*
		1-yr	3-yr	5-yr	*P*	1-yr	*3*-yr	5-yrs	
Gender									
Male	240	41.7%	29.2%	25.3%		66.7%	43.8%	37.1%	
Female	31	54.8%	38.7%	38.7%	0.095	83.9%	61.3%	54.8%	0.034
Age (years)									
≤ 50	131	41.2%	31.3%	28.2%		67.2%	45.0%	37.4%	
> 50	140	48.6%	29.3%	25.4%	0.565	70.0%	46.4%	40.7%	0.619
HCC family history									
Yes	18	50.0%	27.8%	22.2%		83.3%	55.6%	55.6%	
No	253	42.7%	30.4%	27.1%	0.864	67.6%	45.1%	37.9%	0.148
HBsAg									
Negative	32	59.4%	37.5%	37.5%		78.1%	50.0%	43.8%	
Positive	239	41.0%	29.3%	25.3%	0.148	67.4%	45.2%	38.5%	0.366
Child-pugh stage									
A	269	43.1%	30.1%	26.6%		68.8%	45.7%	39.0%	
B	2	50.0%	50.0%	50.0%	0.485	50.0%	50.0%	50.0%	0.799
AFP (ng/ml)									
≤ 20	67	55.2%	35.8%	34.3%		73.1%	52.2%	47.8%	
> 20	204	39.2%	28.4%	24.3%	0.078	67.2%	43.6%	36.2%	0.063
Edmondson grading									
I-II	212	47.6%	34.4%	30.5%		71.7%	49.5%	43.8%	
III-IV	59	27.1%	15.3%	13.6%	< 0.001	57.6%	32.2%	22.0%	0.002
Tumor size (cm)									
≤ 5	96	66.7%	50.0%	42.4%		87.5%	67.7%	60.4%	
> 5	175	29.7%	19.4%	18.3%	< 0.001	58.3%	33.7%	27.4%	< 0.001
Liver Cirrhosis									
Absent	55	41.8%	30.9%	27.3%		76.4%	47.3%	41.8%	
Present	216	43.5%	30.1%	26.7%	0.973	66.7%	45.4%	38.4%	0.399
Capsulation									
Capsulated	171	53.8%	37.4%	35.6%		78.4%	55.0%	49.1%	
Non-caspulated	100	25.0%	18.0%	11.6%	< 0.001	52.0%	30.0%	22.0%	< 0.001
Tumor number									
Single	185	51.9%	38.4%	34.0%		75.7%	55.1%	49.7%	
Multiple	86	24.4%	12.7%	11.1%	< 0.001	53.5%	25.6%	16.3%	< 0.001
Vascular invasion									
Yes	56	12.5%	7.1%	5.4%		32.1%	16.1%	8.9%	
No	215	51.2%	36.3%	32.4%	< 0.001	78.1%	53.5%	47.0%	< 0.001
HRNR expression									
Low	114	61.4%	49.1%	45.3%		78.9%	65.8%	63.1%	
High	157	29.9%	16.6%	13.4%	< 0.001	61.1%	31.2%	21.6%	< 0.001
TNM stage									
I-II	152	59.9%	44.7%	40.6%		84.9%	63.2%	57.9%	
III-IV	119	21.8%	11.8%	9.2%	< 0.001	47.9%	23.5%	15.1%	< 0.001

*DFS* disease-free survival, *OS* overall survival. Other abbreviations as in Table 1

**Table 3:** Prognostic factors for DFS and OS by multivariate Cox Proportional Hazards Regression Model

Variables	DFS			OS		
	HR	95%CI	*P*	HR	95%CI	*P*
Tumor size (cm) (> 5 vs ≤ 5)	1.849	1.343–2.546	< 0.001	1.829	1.276–2.621	0.001
Capsulation (capsulated vs non-caspulated)	0.621	0.458–0.842	0.002	0.644	0.461–0.899	0.010
Tumor number (single vs mulitiple)	0.614	0.456–0.828	0.001	0.607	0.440–0.838	0.002
Vascular invasion (Yes vs No)	1.817	1.274–2.590	0.001	1.691	1.156–2.474	0.007
HRNR expression (High vs Low)	2.209	1.627–2.998	< 0.001	2.459	1.736–3.484	< 0.001

*HR* hazard ratio, *CI* confidence interval. Other abbreviations as in Table 1

### HRNR enhances proliferation, colony formation, migration and invasion of HCC cells

**Fig. 3:**
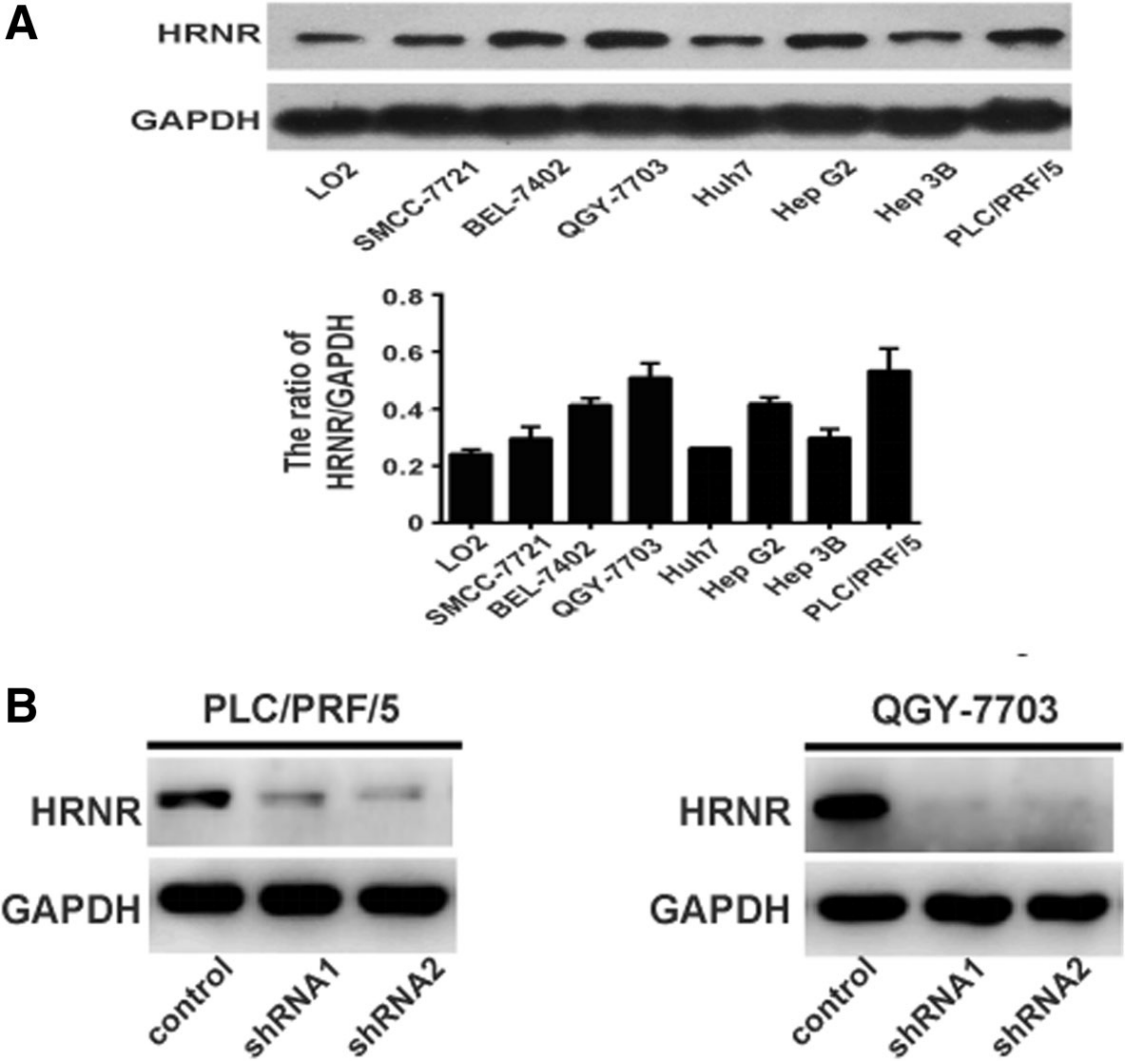
HRNR expression in HCC cell lines. **a** Western blot analysis of HRNR expression levels in a panel of HCC cell lines. **b** HRNR shRNAs inhibited the expression of HRNR in PLC/PRF/5 and QGY-7703 cells

**Fig. 4:**
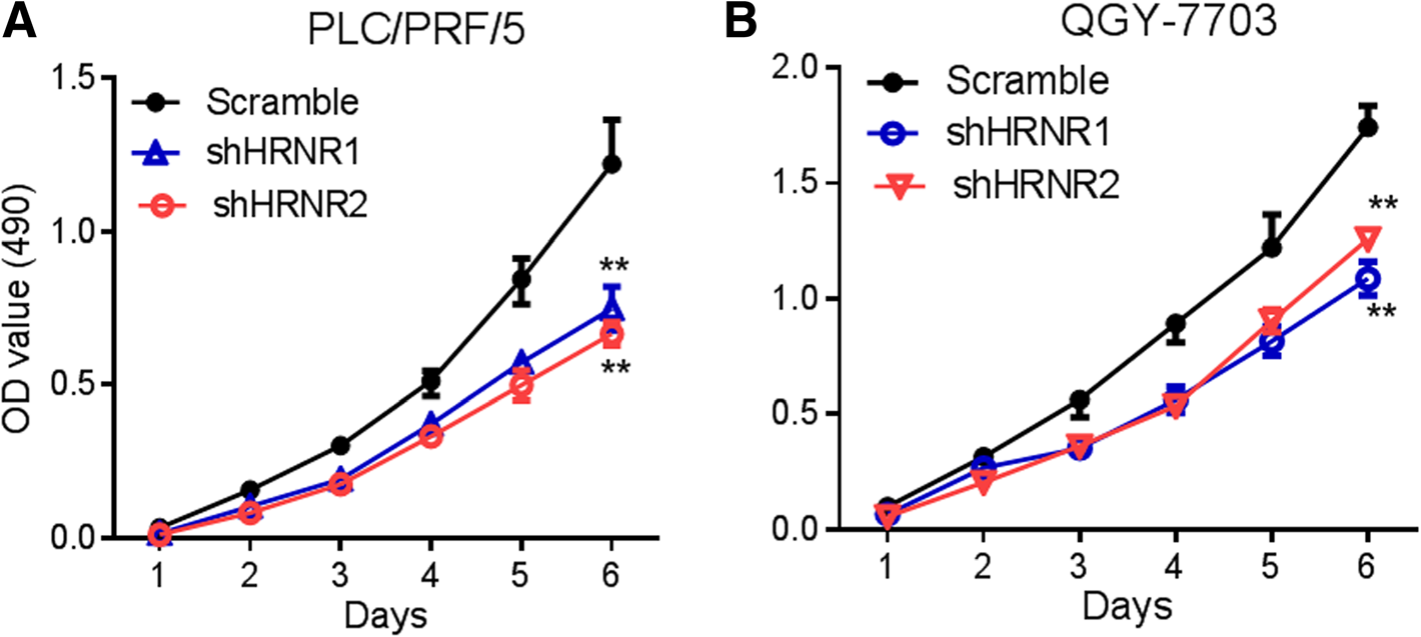
The effect of HRNR on proliferation. The proliferation was measured by the MTT assay, when HRNR expression was knocked down by HRNR- shRNAs in (**a**) PLC/PRF/5 and (**b**) QGY-7703 cells

To investigate the roles of HRNR in HCC progression, we first detected the expression levels of HRNR in different HCC cell lines. The result indicated that the expression levels of HRNR were different in HCC cell lines, with the highest expression detected in PLC/PRF/5 and QGY-7703 cell lines (Fig. 3a). Thus, we selected these two cell lines for further analysis. We determined whether reducing HRNR expression ameliorated tumor growth. We knocked down HRNR expression in PLC/PRF/5 and QGY-7703 cell lines using two independent shRNA constructs (Fig. 3b). The proliferation assay showed that when HRNR expression was knocked down by HRNR-shRNAs in PLC/PRF/5 cells, tumor cells proliferation was suppressed compared with the PLC/PRF/5 scramble control cells (*P* <  0.01). Similarly, the proliferation of QGY-7703-shRNA1-HRNR and QGY-7703- shRNA2-HRNR cells was also significantly decreased (*P* <  0.01) (Fig. 4).

**Fig. 5:**
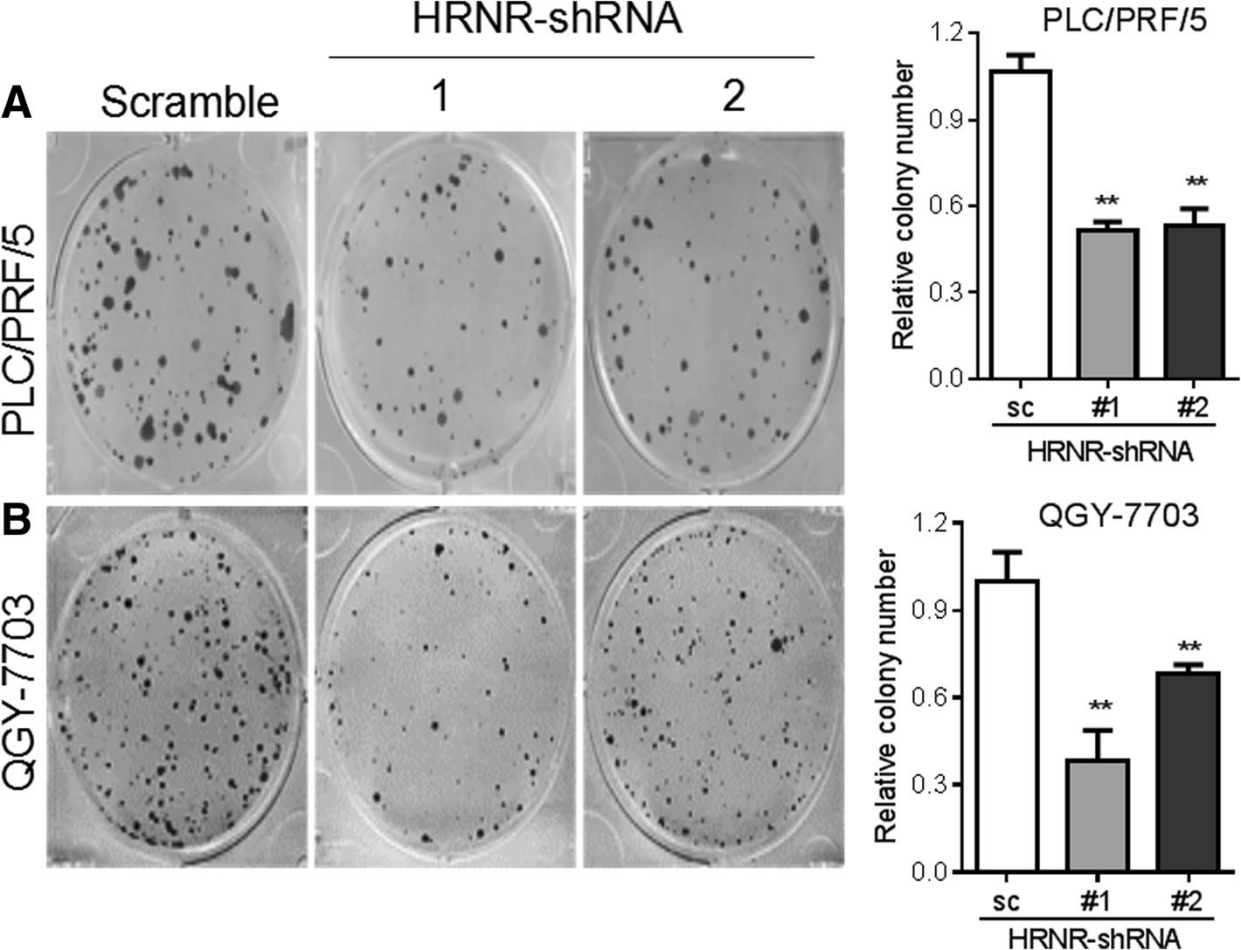
Silencing of HRNR with shRNAs inhibits colony formation of HCC cells. Colony formation assays of (**a**) PLC/PRF/5 and (**b**) QGY-7703 cells when HRNR was knocked down with shRNAs

**Fig. 6:**
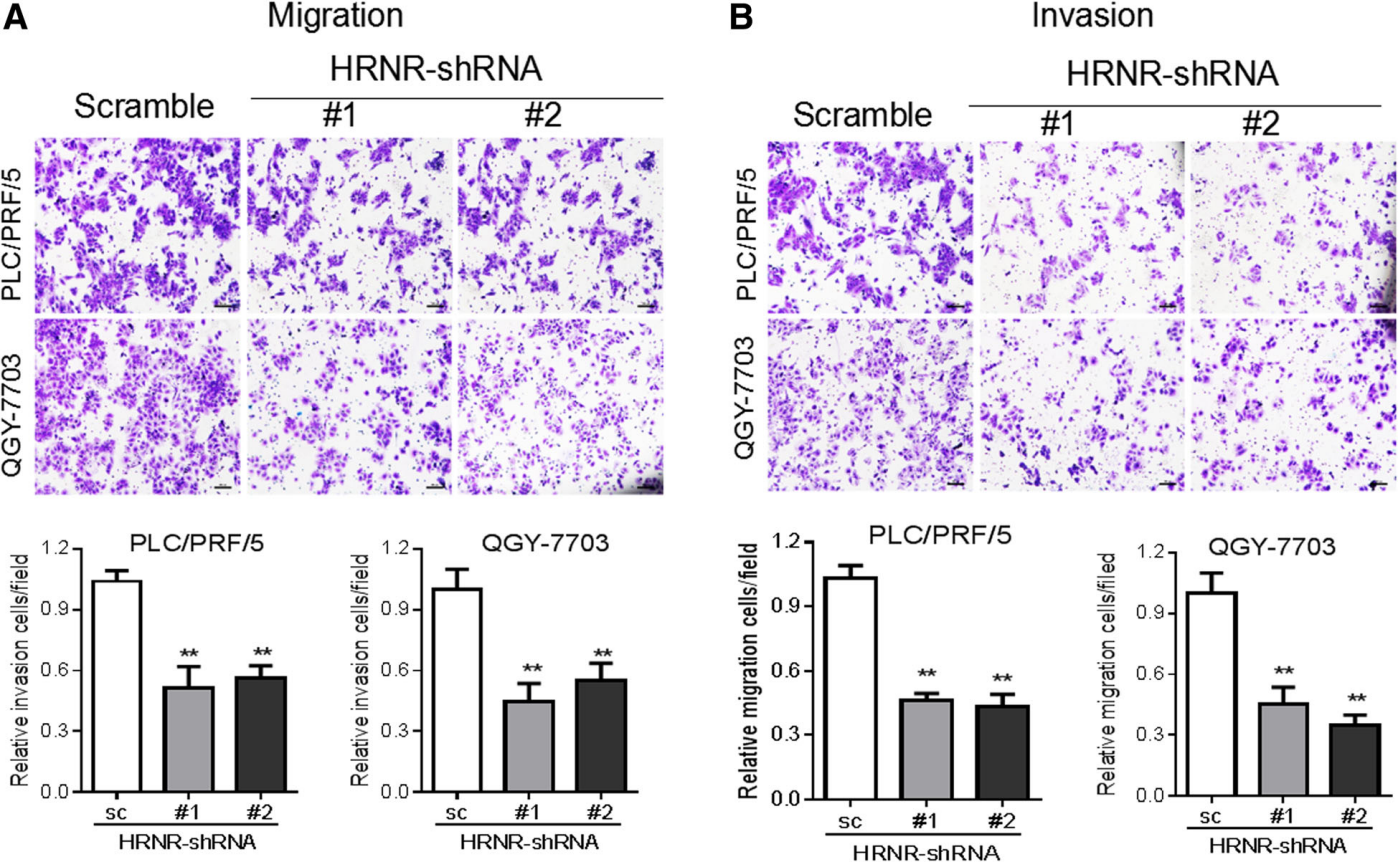
HRNR enhances tumor cell migration and invasion. Transwell assays of the ability of HRNR in (**a**) migration and (**b**) invasion in PLC/PRF/5 and QGY-7703 cells when HRNR was knocked down with shRNAs

We next determined the functional role of HRNR in aggressive growth properties of tumor cells by performing colony formation and migration assays. Our results showed that the silencing of HRNR with shRNA1 and shRNA2 inhibited colony formation in PLC/PRF/5 cells compared to control cells (*P* <  0.01). The same phenomena were also observed in QGY-7703 cells (*P* <  0.01) (Fig. 5). The transwell migration assay revealed an important suppression of cell migration in PLC/PRF/5-shRNA1-HRNR and PLC/PRF/5-shRNA2-HRNR cells compared with the PLC/PRF/5-scramble control cells. Similarly, when compared to the QGY-7703-scramble control cells, the migration was less in both QGY-7703-shRNA1-HRNR and QGY-7703-shRNA2-HRNR cells (Fig. 6a). Moreover, the invasion assays demonstrated that knocking down HRNR significantly impaired the invasiveness of both PLC/PRF/5 and QGY-7703 tumor cells (Fig. 6b).

### HRNR promotes HCC tumor growth in vivo

**Fig. 7:**
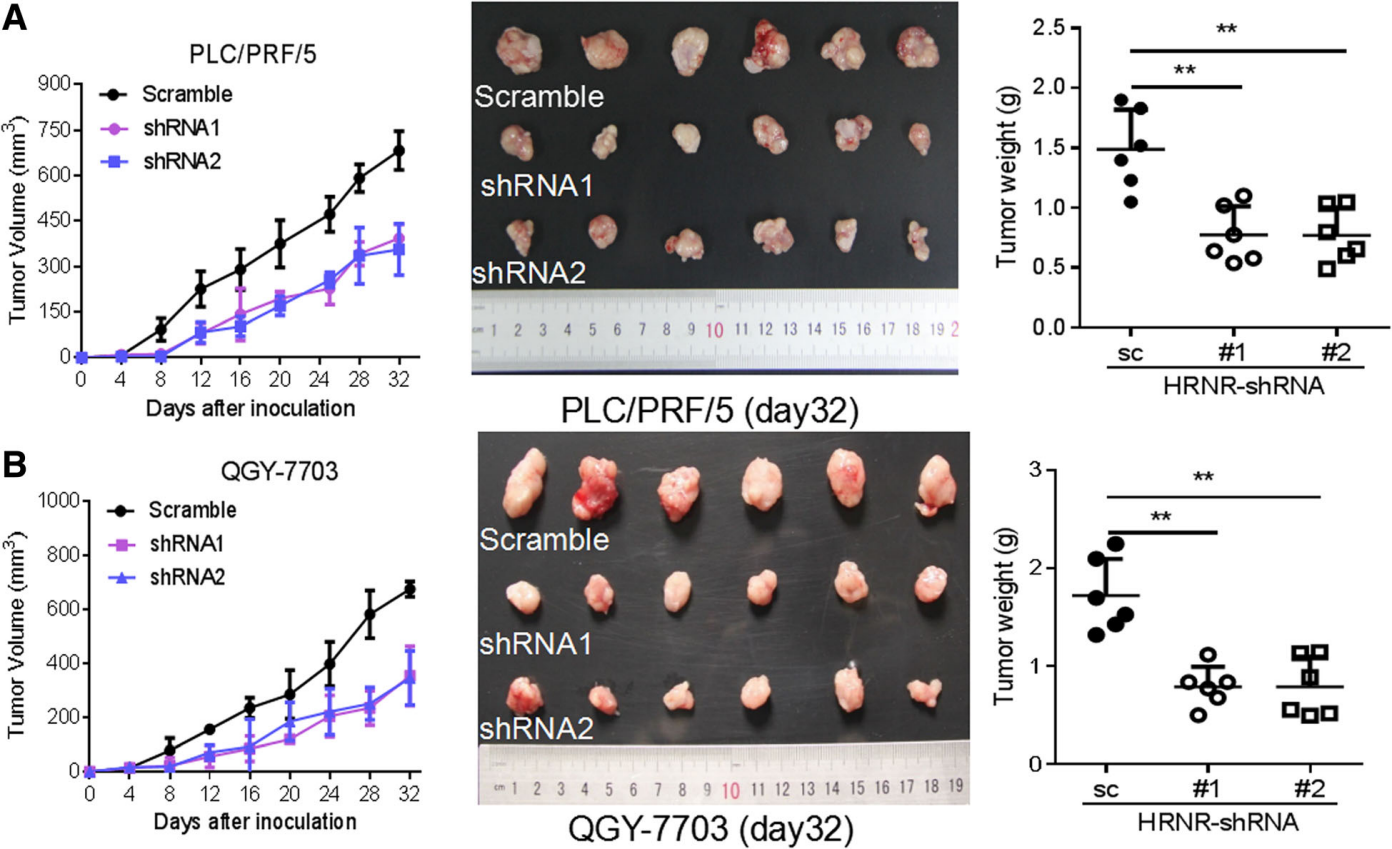
HRNR promotes HCC growth in vivo. BALB/C nude mice (*n* = 6) were injected with (**a**) PLC/PRF/5-scramble, PLC/PRF/5-shRNA1-HRNR and PLC/PRF/5-shRNA2-HRNR cells; (**b**) QGY-7703-scramble, QGY-7703-shRNA1-HRNR and QGY-7703-shRNA2-HRNR cells. Tumor growth was monitored. Mice were sacrificed on day 32 post-injection; tumors were harvested, and weighted

To further explore the biological importance of HRNR in HCC, we examined the tumor growth in xenograft experiments. Human tumor cells were injected subcutaneously in nude mice and tumor growth was monitored. As represented in Fig. 7a, tumor growth in mice injected with PLC/PRF/5-shRNA1-HRNR and PLC/PRF/5-shRNA2-HRNR cells was significantly decreased compared with the control group. Furthermore, tumor weight was positively associated with the expression levels of HRNR. We also found that inhibiting HRNR reduced tumour growth in the xenograft model with QGY-7703 tumour cells (Fig. 7b). Collectively, these data suggest that HRNR plays a critical role in HCC tumor growth in vivo.

### Loss of HRNR inhibits the phosphorylation of AKT in HCC cells

**Fig. 8:**
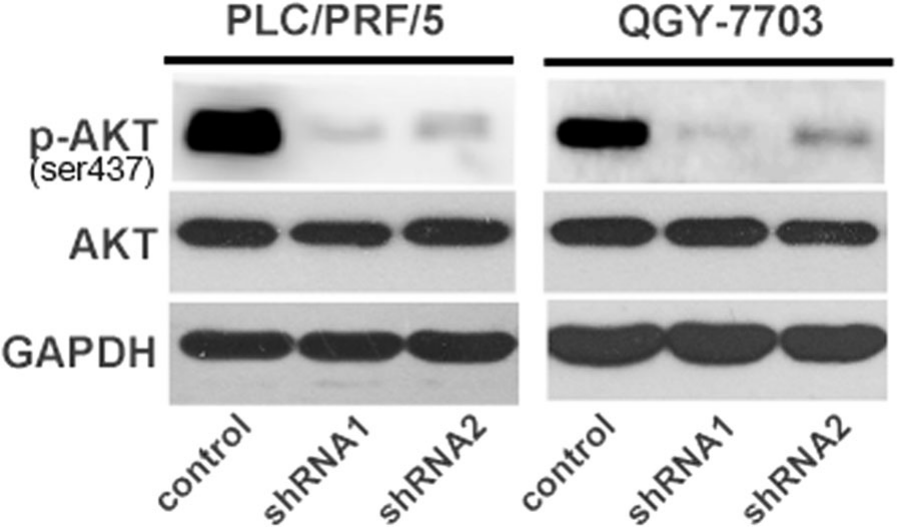
HRNR influences AKT phosphorylation in HCC cells. Western Blot analysis of AKT phosphorylation and total AKT expression in PLC/PRF/5-shRNA-HRNR, QGY-7703-shRNA-HRNR and control cells

Finally, we explored the potential mechanism responsible for HRNR-mediated tumor growth. HRNR is a member of the S100 protein family. Emerging evidence has indicated that the functional role of S100 protein family members, such as S100A1, A100A4 and S100A16, is closely associated with AKT phosphorylation and activation [18–20]. We proposed that the role of HRNR in HCC might also be through regulating AKT phosphorylation. To test this, we analyzed AKT expression and phosphorylation by western blot. We found that AKT phosphorylation was suppressed after knockdown of HRNA in PLC/PRF/5 and QGY-7703 cells, whereas the expression level of total AKT was not changed (Fig. 8). Together, our results imply that HRNR may promote HCC via AKT phosphorylation.

## Discussion

In the study, we firstly detected HRNR expression in 271 HCC samples and HCC cell lines, and found that HRNR was frequently up-regulated in HCC tissues and cells. Second, we explored the prognostic value of HRNR expression in HCC patients after liver resection. We verified the clinical importance of HRNR as an independent prognostic indicator for HCC patients after hepatectomy. These results suggested that HRNR might play a vital role in cancer progression. Therefore, we investigated how HRNR contributed to the progression of HCC. We found that HRNR enhanced cell proliferation and colony formation as well as migration and invasion in vitro and tumor growth in vivo.

The S100 protein family, with over 20 members, is the largest subgroup of calcium binding proteins. The proteins in this family have amino acid sequence similarity as well as the functional EF-hand structure motif, which plays a vital role in calcium binding via a helix-loop-helix topology [21]. Proteins containing this motif are taken in various pathological and physiological cell functions [22–24]. As a member of the S100 protein family, the role of HRNR still remains to be fully understood, especially in cancer research. The expression of HRNR was found in breast epithelial cells, macrophages and stromal fibroblasts. The unique regulation of HRNR expression was found in different stages of mammary development. The expression levels of HRNR were increased in invasive lobular carcinomas and less aggressive breast carcinoma compared to invasive ductal carcinomas phenotypes. During the induction of apoptosis, the expression levels of HRNR were altered [25]. Choi et al. demonstrated that HRNR was included in breast cancer development and malignant conversion from preinvasive lesions [8]. Our results demonstrated that HRNR promoted tumor progression and was connected with poor prognosis for HCC.

The activation of AKT kinase is essential for metastatic pathways, containing the escape of tumor cells from the tumor microenvironment, migration into and then out of the circulation, stimulation of angiogenesis, obstruction of apoptosis, and initiation of proliferation [26, 27]. A series of processes in metastasis are regulated by the activation of AKT via phosphorylation at Thr-308 by PDK1 and Ser-473 by a complex involving mammalian/mechanistic target rapamycin/Rictor (mTORC2) [26, 28]. AKT phosphorylates many cellular proteins, containing GSK3α, GSK3β, BAD, and p27KIP1 to promote survival and cell cycle [29]. In addition, AKT phosphorylates and inactivates Tuberin, a GTPase-activating protein (GAP) for the Ras homologue Rheb. Inactivation of Tuberin permits GTP-bound Rheb to gather and activate the mammalian/mechanistic target rapamycin//Raptor (mTORC1) complex, which finally regulates protein synthesis, RNA translation, cell growth, and autophagy [30]. We have also provided evidence suggesting that HRNR signals through the AKT cascade to regulate cancer cell behavior; however, how HRNR links to AKT activation remains to be determined. More investigation is needed to delineate the signaling mechanism underlying the AKT activation by HRNR.

## Conclusions

Our results demonstrated that HRNR, which is frequently overexpressed in HCC, was linked with aggressive tumor phenotypes and poor prognosis for HCC patients after liver resection. In addition, the in vitro and in vivo assays validated the promoting role of HRNR in HCC progression. Further, we demonstrated that the loss of HRNR inhibited the phosphorylation of AKT in HCC cells. Therefore, we propose that strategies designed to downregulate HRNR in HCC patients with high HRNR expression may provide a promising approach to alleviate HCC progression.

## Abbreviations

CI: Confidence internal
DFS: Disease-free survival
DMEM: Dulbecco’s modified Eagle media
ECL: Electrochemical luminescence
FBS: Fetal bovine serum
GAP: GTPase-activating protein
HCC: Hepatocellular carcinoma
HR: Hazard risk
HRNR: Hornerin
IHC: Immunohistochemical
mTORC1: mammalian/mechanistic target rapamycin/Raptor
mTORC2: mammalian/mechanistic target rapamycin/Rictor
OS: Overall survival
PBS: Phosphatebuffered saline
TNM: Tumor-node-metastasis
